# Cystic Tumor of Conjunctiva Produced by Striking the Eye with Bristles of a Hair Brush

**Published:** 1889-12

**Authors:** S. Latimer Phillips

**Affiliations:** Savannah, Ga.


### CYSTIC TUMOR OF CONJUNCTIVA PRODUCED BY STRIKING THE EYE WITH BRISTLES OF A HAIR BRUSH.

BY S. LATIMER PHILLIPS, M. D., SAVANNAH, GA.

S. W. W., æt. 28, mulatto, consulted me for a painful eye, giving the following history:

Three weeks before visiting me had, while in the barber's chair, been brushed accidentally in the right eye by hair brush in the hand of the barber. At this time there was but little pain. Three days after accident began to have pain in eye which gradually grew worse, and finally became so bad he had to stop work and seek relief. The pain was always worse at night, and so severe that he was able to get but little sleep. At time pain developed he noticed a small red bump on the eye, which has been slowly increasing in size. One week before I saw him he noticed that the bump had become yellow.

When I saw patient for first time, there was much photophobia and lachrymation, v. 20–46 each eye, but could see a little more distinctly out of left than right. There was now a yellowish tumor the size of a split garden pea, and resembling it much in color and shape, under the conjunctiva of the outer side of the cornea about three lines away. It was movable to a degree, and the vessels of the conjunctiva were running to it from every direction, greatly engorged, and the conjunctiva in this region much swollen. Such a condition we see in ordinary phlyctenular conjunctivitis. The eye was freely movable in every direction, but it was painful to do so. In lower lid of same side, showing through skin, near tarsal border, was another yellow swelling of same size, but not painful, and this he had had for a longer time. This was opened and proved to be a suppurating tarsal cyst. Thinking from the history, the appearance and the abscess in the lower lid, it might be an abscess also of the conjunctiva, a small Graefe's knife was passed into it from the temporal side, the eye being under the influence of cocaine, but on withdrawing the knife nothing came out. There was some little bleeding. The eye was dressed with a compress and bandage, and cocaine two per cent., to be used in case of pain. On the next day swelling was reduced some in size, and much of the yellow color gone. No pain. It was again punctured with a similar result; the eye still remained conjested.

Two days after the tumor was still apparent, though smaller. A free opening was made which was followed by the escape of some glutinous water. Cocaine was still used two or three times per day, with compress for a few days, after which he was able to resume his work. A week later I saw him; he had discontinued treatment, and naught was seen but a small brown pigmented patch under the conjunctiva where the tumor had been.

Salezowski * mentions three kinds of conjunctival cysts; the transparent like vesicles situated in the ocular conjunctiva; the white, sebaceous confined to the palpebral conjunctiva, usually; and the hydatids in both ocular and palpebral. DeWecker† says "the conjunctival cysts constitute a variety of benign tumors which are observed on very rare occasions." They are usually situated near the border of the cornea, and vary from the size of a large pin head to that of a pea. They are round or oval, and circumscribed. Sometimes the color is a rose, sometimes transparent, and again yellow. Their membrane envelope is more or less resistant, while the contents may be liquid or thickish. Meyer‡. According to Arlt§ simple serous cysts are seen after blows on the eye. Others also believe them to come after trauma. (Zaunder and Seissler, Ubthoff.) But according to DeWecker || we have no positive knowledge of the cause. Schmidt-Rimpler¶ observed a case developed on the apex of a pteryginm. The only treatment is to open the cyst freely and let out its contents; if this is not sufficient, the cyst walls will have to be destroyed by slight cauterization with stick nitrate of silver; many authors advise complete enucleation of the cyst, but owing to the thinness of the walls, it is difficult to do, the walls usually giving way during the operation.

*Salezowski. Traite des Maladies des Yeux. Paris, 1888. P. 233.

† DeWecker, Therapeutique Oculaire, Paris, 1878. P. 143.

‡Meyer A Practical Treatise on Diseases of the Eye. Translated from the French by Freeland Fergus, Philadelphia, 1887. P. 111.

§ Arlt. Chemical Studies on Diseases of the Eye. Translated from the German by Lyman Ware. Philadelphia, 1885. P. 112.

||Op. cit., p. 143.

¶Schmidt-Rimpler. Augenheilkunde und Ophthalmoscopie. Berlin, 1888. P. 451.

