# Errata

**Published:** 1890-02

**Authors:** 


ERRATA.—In the December number the following errors were made in Dr. S. Latimer Phillips' article:

On page 589, line 14 from top, " v. $\frac{20}{46}$ " should read v. $\frac{20}{20}$.

On page 590, last word on line 8 from top, should read congested instead of " conjested."

Line 11, on same page, " water " should read matter.

Line 17, on same page, " Salezowski " should read Galezowski.

Same correction in note at bottom of page.

Line 10 from bottom of same page, " Zaunder " should read Zauder.

Line 9 from bottom of same page, " Seissler, Ubthoff " should read Seissler Ubthoff.

Line 7 from bottom of same page, " pteryginum " should read pterygium.

Note 4 at bottom of same page, " Chemical " should read Clinical.

